# Physiological characteristics and virulence gene composition of selected serovars of seafood-borne *Salmonella enterica*

**DOI:** 10.14202/vetworld.2023.431-438

**Published:** 2023-03-15

**Authors:** Fathima Salam, Manjusha Lekshmi, Parmanand Prabhakar, Sanath H. Kumar, Binaya Bhusan Nayak

**Affiliations:** 1 Quality Control Laboratory, ICAR-Central Institute of Fisheries Education, Mumbai, Maharashtra, India; 2 Fish Processing Technology, College of Fisheries, Bihar Animal Sciences University, Patna, Bihar, India

**Keywords:** biofilm, invasion, non-typhoidal *Salmonella*, *Salmonella* pathogenicity islands, seafood, virulence

## Abstract

**Background and Aim:**

All serotypes of *Salmonella enterica* are considered potentially pathogenic. However, the non-typhoidal *Salmonella* (NTS) serotypes vary considerably in terms of pathogenicity and the severity of infections. Although diverse serotypes of NTS have been reported from tropical seafood, their sources, physiological characteristics, and virulence potentials are not well understood. This study aimed to compare the physiological characteristics of selected serovars of *Salmonella* from seafood and investigate possible variations in the distribution of known genes within the pathogenicity islands.

**Materials and Methods:**

A series of biochemical tests, including carbohydrate fermentation and amino acid decarboxylation tests were performed to physiologically compare the isolates. The genetic characterization with respect to putative virulence genes was done by screening for genes associated with *Salmonella* pathogenicity island (SPI) I–V, as well as the toxin- and prophage-associated genes by polymerase chain reaction.

**Results:**

Irrespective of serotypes, all the isolates uniformly harbored the five SPIs screened in this study. However, some virulence genes, such as the *avrA*, *sodC*, and *gogB* were not detected in all *Salmonella* isolates. The biochemical profiles of *Salmonella* serotypes were highly conserved except for variations in inositol fermentation and citrate utilization. All the isolates of this study were weak biofilm formers on polystyrene surfaces.

**Conclusion:**

The pathogenicity profiles of environmental NTS isolates observed in this study suggest that they possess the virulence machinery necessary to cause human infections and therefore, urgent measures to contain *Salmonella* contamination of seafood are required to ensure the safety of consumers.

## Introduction

Salmonellosis is one of the most common food-borne diseases globally, accounting for 93.8 million food-borne illnesses and 155,000 deaths/year worldwide [1]. *Salmonella* is gram-negative, rod-shaped, non-spore forming, facultatively anaerobic bacteria belonging to the family *Enterobacteriaceae*, and most members of the genus are motile with peritrichous flagella. The majority of *Salmonella* are hydrogen sulfide producers, lactose non-fermenters, catalase positive, and oxidase negative. Salmonellae are primary inhabitants of the gastrointestinal tract of a wide range of food animals, wild animals, rodents, birds, reptiles, and insects, usually without the display of any apparent illness [2]. They can be disseminated through feces to the soil, water, foods and feeds, and other animals, including humans. Although *Salmonella* is not a natural inhabitant of the aquatic environment, diverse *Salmonella* serovars are widely distributed in fresh and coastal-marine water and a variety of seafood, including mollusks, shrimp, clams, and various fish species [3, 4]. Unhygienic handling and poor sanitation in landing centers and retail markets contribute to post-harvest contamination of seafood [5].

The ability of Salmonellae to colonize the host and cause infection is associated with different gene clusters known as the *Salmonella* pathogenicity islands (SPIs) located on the chromosome and the virulence plasmids [6]. Each SPI is responsible for various cellular activities resulting in the expression of virulence by the organism [6]. SPIs are segments of DNA speculated to have been obtained from other bacteria by horizontal gene transfer (HGT) events. To date, 23 SPIs have been identified although the functions in the pathogenesis of *Salmonella* and virulence of those genes contained within each island have not yet been completely described [7]. Five of these SPIs (SPI-1 to SPI-5) are well characterized [8]. Differences in the presence of genes of SPIs are responsible for the differences in the pathogenicity of non-typhoidal *Salmonella*. For example, *Salmonella* Kentucky with a truncated SPI-2 that is devoid of certain essential virulence and fimbrial genes, is probably less able to infect humans [7].

SPI-1 is a conserved 40 kb genomic island found consisting of a type 3 secretion system (T3SS-1), its effector proteins and several regulatory genes essential for the invasion of the host epithelial cells [6, 9]. SPI-2 encodes for a second secretion system (T3SS-2) which works in tandem with T3SS-1 [10]. SPI-3 contains ten open reading frames (ORFs) constituting six transcriptional units. The major virulence gene *mgtCB* is required for the survival of *Salmonella* in macrophages and low Mg^2+^ environments [11]. SPI-4 encodes a type I secretion system that contributes to the adhesion of *Salmonella* to epithelial cells, while SPI-5 plays a crucial role in enteropathogenicity and encodes different genes associated with intestinal mucosal fluid secretion and inflammatory responses [12].

*Salmonella* can form biofilms on both biotic and abiotic surfaces, which promotes survival under low nutrient conditions, heat and acidic pH, low temperature, disinfectants and antimicrobials, and biofilms can also be important sources of food contamination in processing facilities [13]. The ability of *Salmonella* to form biofilm depends on various factors such as the growth phase of the cell, growth medium, contact time, type and properties of the inert material, and environmental parameters such as pH, temperature, and presence of organic material [14].

*Salmonella* is frequently encountered in tropical seafood due to fecal contamination of coastal water and poor sanitation in markets and landing centers. Diverse NTS serovars isolated from seafood and the environment constitute a food safety concern, although the true pathogenicity of these serovars is not investigated in detail. The contamination of seafood can occur from diverse sources of *Salmonella* with varying potentials to cause human infections. This study aimed to investigate these possible differences by comparing the physiological and virulence characteristics of selected Salmonella serovars isolated from seafood.

## Materials and Methods

### Ethical approval

No ethical approval was required for this study.

### Study period and location

This study was conducted from February to October 2021 at the Quality Control Laboratory, ICAR-Central Institute of Fisheries Education, Mumbai, Maharashtra. The *Salmonella* serotypes used in the study were previously isolated from fish and shellfish samples sold in the retail markets and fish landing centers in and around Versova, Mumbai, Maharashtra, India [4].

### Bacterial strains

Eight isolates of *Salmonella enterica* serovars consisting of *Salmonella* Typhimurium (5), *Salmonella* Tennessee (2), and *Salmonella* Lindenberg (1) were used (Table-1). *Salmonella* Typhimurium ATCC 14028 was used as the reference strain in all experiments. The frozen glycerol stocks were retrieved by inoculating into Luria Bertani (LB) broth, followed by isolation on LB agar.

**Table-1:** Details of *Salmonella* isolates used in this study.

Isolate No.	Serotype	Source^ψ^
329	*S*. Typhimurium	*Harpadon nehereus*
331	*S.* Typhimurium	*Harpadon nehereus*
332	*S.* Typhimurium	*Harpadon nehereus*
333	*S.* Typhimurium	*Harpadon nehereus*
334	*S*. Typhimurium	*Harpadon nehereus*
342	*S.* Tennessee	*Dicentrarchus labrax*
343	*S*. Tennessee	*Dicentrarchus labrax*
346	*S*. Lindenberg	*Dicentrarchus labrax*
ATCC 14028	*S*. Typhimurium	-

ψ All finfish species are of marine origin sampled from the retail markets. *S*. Typhimurium=*Salmonella* Typhimurium, *S.* Tennessee=*Salmonella* Tennessee, *S*. Lindenberg=*Salmonella* Lindenberg

### Physiological characterization

The physiological characteristics of the *Salmonella* serovars were studied using a total of 29 biochemical tests, comprising of triple sugar iron (TSI), lysine iron agar (LIA), urease, malonate, indole, methyl red (MR), Voges-Proskauer (VP), citrate, carbohydrate utilization, and amino acid decarboxylase tests [15]. Biofilm forming ability of the test isolates was analyzed at different temperatures of 12°C, 37°C, and 43°C for incubation periods of 24 h and 48 h following the protocol described by O’Toole and Kolter [16]. Briefly, the overnight cultures of bacteria were diluted (1:100) in tryptic soy broth (TSB) and 100 μL of the suspension was dispensed into each of 3 wells of a microtiter plate with fresh TSB medium as blank. The plates were incubated at different temperatures of 12°C, 37°C, and 43°C for 24 h and 48 h. After incubation, the optical density (OD) of growth was measured at 595 nm. Non-adherent bacteria were removed, and the plates were washed three times with distilled water and stained using 125 μL of 0.1% freshly prepared crystal violet for 20 min at room temperature. After discarding the staining liquid, loosely bound bacteria and dye were removed from the wells by washing three times with 125 μL distilled water, followed by air drying and destaining with 200 μL of 95% ethanol at room temperature for 45 min. A 100 μL volume of the mixture was transferred to a fresh microtiter plate, and the OD was measured at 595 nm. The categorization of biofilm into strong, moderate, and weak biofilm was done as described previously [17].

### Screening for SPI-associated genes

The distribution of known SPI-associated genes was studied by polymerase chain reaction (PCR) targeting important virulence genes present on five major SPIs and prophages, namely *invA*, *ivnB*, *invC*, *invE*, *invF*, *invG*, *invH*, *invI and invJ*, *hilA*, *avrA*, *iroB* (SPI-1), *spiC*, *ttrC* (SPI-2), *marT*, *mgtC*, *misL* (SPI-3), *orfL* (SPI-4), *sopB*, *pipD* (SPI-5), *Salmonella* enterotoxin (*stn*) (toxin), and *gogB* and *sodC* (prophage). All amplifications were carried in 30 mL reaction volumes consisting of a 10× buffer, 30 picomoles of forward and reverse primers, 2.5 mM concentrations of each of the four deoxynucleoside triphosphates, and 1 U of *Taq* DNA polymerase (Takara, Japan). The oligonucleotide sequences are shown in Table-2 [18–24]. PCR amplifications using primers designed in this study were done at 94°C for 5 min, followed by 30 cycles of 94°C for 1 min, 55°C for 1 min and 72°C for 1 min, and a final extension at 72°C for 5 min. All PCR amplifications were carried out in a SimpliAmp thermal cycler (Thermo Fisher Scientific, USA). The amplified DNA products were confirmed by 1%–2% (w/v) agarose gel electrophoresis and photographed using a gel documentation system (Bio-Rad, Hercules, CA, USA).

**Table-2:** Oligonucleotide primers used for PCR detection of virulence-associated genes of *Salmonella*.

Primers	SPI	Sequence 5’–3’	Amplicon size (bp)	Reference
*invA*	SPI-1	F-ctcgcctttgctggttttag R-gccatggtatggatttgtcc	347	This study
*invB*	SPI-1	F-aaaatccggatgcactaagg R-attagttcgttccgcactgg	301	This study
*invC*	SPI-1	F-gaaatcggaagtggcaaaaa R-gttgagcgttttacccctga	348	This study
*invE*	SPI-1	F-tgcaatagcgaaagcatcag R-tgtgttacgcgaattgcttc	495	This study
*invF*	SPI-1	F-ctgcacaaacgacgaaaatg R-ctgaaagccgacacaatgaa	430	This study
*invG*	SPI-1	F-cagacgcatcacccctattt R-atggcgctacagctaaagga	411	This study
*invH*	SPI-1	F-aactggccaacgcaaatagt R-atgggcagcaagtaacgtct	311	This study
*invI*	SPI-1	F-ctgctgtatctctcgctgga R-ggcgctgtacggtatttcat	380	This study
*invJ*	SPI-1	F-catgcaaacgatgttcaacc R-ccaataccggtaagcctgaa	309	This study
*hilA*	SPI-1	F-ttaaacatgtcgcccaaacagc R-gcaaactcccgatgtat	216	[19]
*avrA*	SPI-1	F-gagctgctttggtcctcaac R-tgttgagcgtctggaaagtg	350	This study
*iroB*	SPI-1	F-tgcgtattctgtttgtcggtcc R-tacgttcccaccattcttccc	606	[20]
*spiC*	SPI-2	F-cctggataatgactattgat R-agtttatggtgattgcgtat	300	[21]
*ttrC*	SPI-2	F-gtgggcggtacaatatttctttt R-tcacgaataataatcagtagcgc	920	[22]
*marT*	SPI-3	F-cggcaaatacgctttatcaga R-cgtcacgcagcctgtatca	536	[23]
*mgtC*	SPI-3	F-tgactatcaatgctccagtgaat R-atttactggccgctatgctgttg	655	[18]
*misL*	SPI-3	F-gtcggcgaatgccgcgaata R-gcgctgttaacgctaatagt	562	[21]
*orfL*	SPI-4	F-ggagtatcgataaagatgtt R-gcgcgtaacgtcagaatcaa	331	[21]
*pipD*	SPI-5	F-cggcgattcatgactttgat R-cgttatcattcggatcgtaa	398	[21]
*sopB*	SPI-5	F-gatgtgattaatgaagaaatgcc R-gcaaaccataaaaactacactca	1170	[22]
*stn*	Enterotoxin	F-ttggtcgtaaaataaggcg R-tgcccaaagcagagattc	267	[24]
*sodC*	Phage-encoded	Fccctgactgtaaagatgaacga R-caatgacaccacaggcaaaa	459	[23]
*gogB*	Phage-encoded	F-attgagaaggaggagcgtaa R-gcttaacataaccatcggtac	721	[23]

PCR=Polymerase chain reaction, SPI=*Salmonella* pathogenicity island

## Results

**Figure-1:**
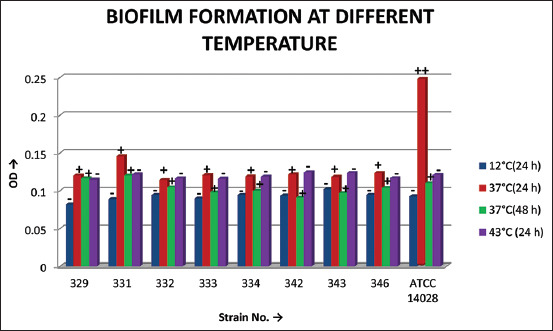
Biofilm formation by different serovars of *Salmonella* tested in this study. The levels of biofilms formed were categorized as no biofilm (−), weak (+), and moderate (++).

A total of 29 biochemical tests were carried out, comprising TSIA, LIA, urease, malonate, indole, MR, VP, citrate (data not shown), carbohydrate utilization, and amino acid decarboxylase tests. The results of carbohydrate utilization and amino acid decarboxylase tests are presented in Tables-3 and 4. The biofilm-forming potential of the isolates was tested and all the isolates of this study were weak biofilm producers at 37°C (Figure-1).

**Table-3:** Carbohydrate utilization profiles of *Salmonella* serovars used in this study.

Carbohydrates	Isolates no.								

	329	331	332	333	334	342	343	346	ATCC 14028
D-Glucose	+	+	+	+	+	+	+	+	+
Lactose	−	−	−	−	−	−	−	−	−
Sucrose	−	−	−	−	−	−	−	−	−
Mannitol	+	+	+	+	+	+	+	+	+
Dulcitol	+	+	+	+	+	+	+	+	-
D-xylose	+	+	+	+	+	+	+	+	+
D-galactose	+	+	+	+	+	+	+	+	+
Inositol	−	−	−	+	−	+	+	+	+
Adonitol	−	−	−	−	−	−	−	−	−
L+Rhamnose	+	+	+	+	+	+	+	+	+
D - Arabinose	−	−	−	−	−	−	−	−	−
D+Raffinose	−	−	−	−	−	−	−	−	−
D+Melibiose	+	+	+	+	+	+	+	+	+
Salicin	−	−	−	−	−	−	−	−	−

Isolates 329, 331, 332, 333 and 334=*Salmonella* Tyhphimurium; Isolates 342 and 343=*Salmonella* Tennessee; Isolate 346=*Salmonella* Lindenberg

**Table-4:** Amino acid decarboxylation activities of *Salmonella* serovars.

Amino acid	329	331	332	333	334	342	343	346	ATCC 14028
Alanine	−	−	−	−	−	−	−	−	−
Methionine	−	−	−	−	−	−	−	−	−
Histidine	+	+	+	+	+	+	+	+	+
Arginine	+	+	+	+	+	+	+	+	+
Ornithine	+	V	+	+	+	+	+	V	+
Lysine	+	V	+	+	+	+	+	+	+

Isolates 329, 331, 332, 333 and 334=*Salmonella* Tyhphimurium; Isolates 342 and 343=*Salmonella* Tennessee; Isolate 346=*Salmonella* Lindenberg. V indicates variable/non-confirmed reaction

All *Salmonella* isolates of this study, irrespective of serovars, were positive for *invA*, *invB*, *invC*, *invE*, *invF*, *invG*, *invH*, *invI*, *invJ*, *hilA*, *iroB*, *sopB*, *spiC*, *ttrC*, *marT*, *mgtC*, *misL*, *orfL*, *pipD*, and *stn*. The *avrA* gene was not detected in *S*. Typhimurium 329, 331, 332, and 334 and the prophage-encoded genes *gogB* and *sod*C were absent in non-Typhimurium serovars of *Salmonella* used in the study.

## Discussion

This study investigated the physiological characteristics and virulence gene composition of seafood-borne *Salmonella* serotypes with respect to the major SPIs. All the test bacterial strains exhibited positive reactions with TSIA, LIA, and MR tests, histidine and arginine decarboxylation and utilized glucose, dextrose, mannitol, dulcitol, xylose, galactose, rhamnose, and melibiose. A negative reaction was observed for urease, malonate, indole, VP tests, alanine and methionine decarboxylation tests and none of the strains utilized lactose, sucrose, adonitol, arabinose, raffinose, and salicin. These results follow the pattern of typical biochemical reactions of *Salmonella* (Tables-3 and 4). *Salmonella* Typhimurium 333 tested negative for citrate utilization. *Salmonella* Typhimurium 329, 331, 332, and 334 were unable to utilize inositol, showing a deviation from the typical *Salmonella* biochemical reactions. A study involving 258 isolates of *Salmonella*. Gallinarum from avian sources in Korea showed a typical pattern of sugar fermentation for lactose, maltose, xylose, sorbitol, galactose, and glucose. In contrast, 95.4% and 93.7% of the isolates showed typical utilization patterns for dulcitol and trehalose, respectively [25]. The prevalence of lactose-fermenting *Salmonella* serovars in different seafood samples has been reported from India, suggesting the possibility of inherent lactose-positive *Salmonella* serovars in fish and shellfish [26]. The utilization of the polyol inositol is facilitated by the *iol* genes, which are located on a 22.6 kb genomic island in *S*. Typhimurium [27]. These authors also suggested, based on the genomic comparison, that inositol utilization genomic island was found in only six *Salmonella* isolates. A protein LolT belonging to the major facilitator superfamily of proteins has been identified as responsible for the transport of inositol in *S*. Typhimurium [28]. The utilization of inositol is also temperature dependent, as 95% of the isolates do not ferment inositol at 37°C, but do so at 25°C [29]. Acquisition or the loss of sugar catabolism genes through the HGT mechanism can also contribute to such variations within a bacterial species, as in the case of sucrose-fermenting strains of *Escherichia coli* [30].

The biofilm-forming ability of the isolates was studied at different temperatures, namely, 12°C, 37°C, and 43°C and two incubation periods of 24 h and 48 h. All the isolates of this study were weak biofilm producers at 37°C (Figure-1). There was not much difference in biofilm formation between 24 h and 48 h of incubation at 37°C. Borges *et al*. [31] studied the biofilm-forming potential of *Salmonella* strains belonging to 11 different serotypes isolated from poultry and foods involved in salmonellosis outbreaks at 3°C, 12°C, 37°C, and 43°C. A total of 243 isolates were tested, and 92.2% (224) of them could produce biofilm under at least one of the tested temperatures, but to varying extents suggesting strain-to-strain variations in biofilm-forming abilities of *Salmonella*. Furthermore, the biofilm-forming ability was not characteristically associated with any specific serovar [31]. Most of the producer strains tested in the study were classified as weak, and just a small number were strong biofilm producers.

**Figure-2:**
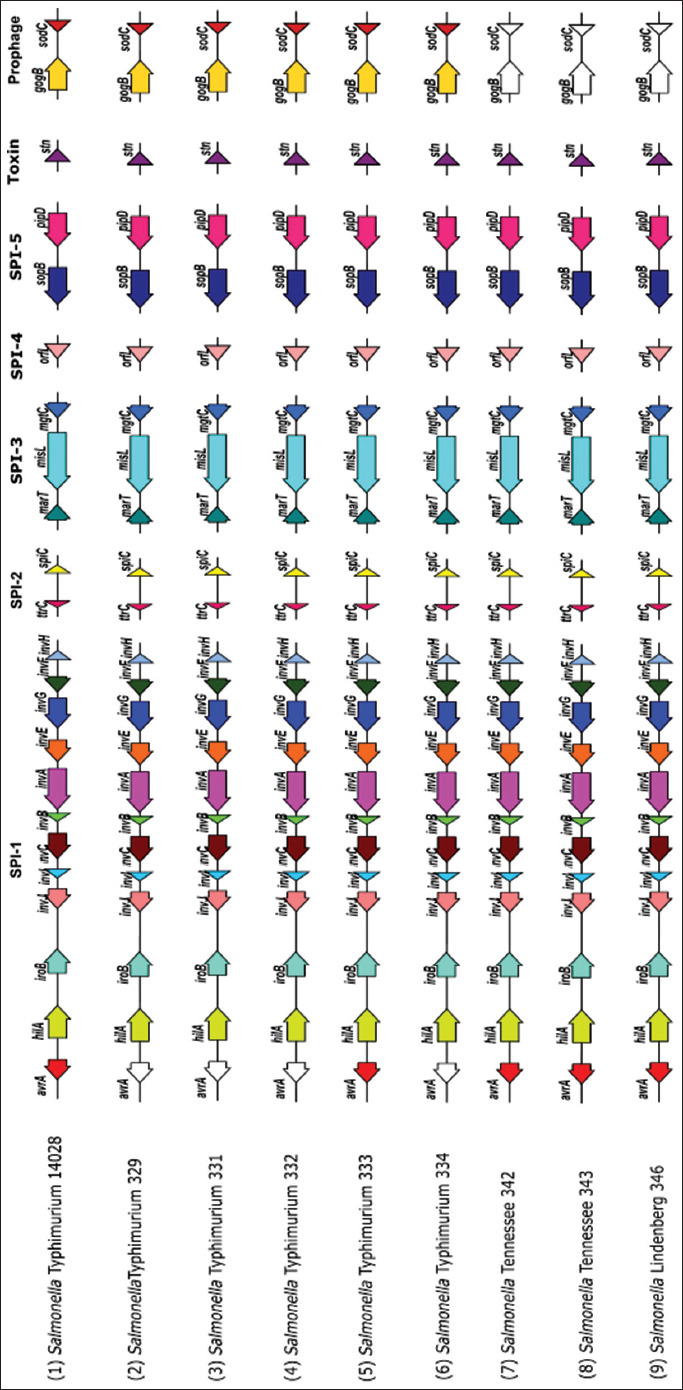
Schematic representation of SPI, toxin and prophage genes of *Salmonella* screened by PCR. Unfilled arrows indicate genes absent from the respective isolate. SPI=*Salmonella* pathogenicity island, PCR=Polymerase chain reaction.

Genetic characterization of the *Salmonella* serovars of this study was done to determine the virulence potentials of environmental serovars by PCR targeting important virulence genes spread over five major SPIs, toxin, and prophages, namely, the *invA*, *hilA*, *iroB*, *avrA* (SPI-1), *spiC*, *ttrC* (SPI-2), *marT*, *mgtC*, *misL* (SPI-3), *orfL* (SPI-4), *sopB, pipD* (SPI-5) *stn* (toxin), and *gogB* and *sodC* (prophage genes). All *Salmonella* isolates of this study, irrespective of serovars, were positive for *invA*, *invB*, *invC*, *invE*, *invF*, *invG*, *invH*, *invI*, *invJ*, *hilA*, *iroB*, *sopB*, *spiC*, *ttrC*, *marT*, *mgtC*, *misL*, *orfL*, *pipD*, and *stn*. The *invA* gene is highly conserved across diverse serovars of *Salmonella*. All (100%) isolates of this study were *invA* positive and also harbored other important virulence genes associated with SPI-1, namely, *invB*, *invC*, *invE*, *invF*, *invG*, *invH*, *invI*, *invJ*, *hilA*, and *iroB* (Figure-2). It is interesting to note that same or different serotypes isolated from different seafood samples are uniform with respect to their invasion gene composition. In contrast, a previous study screening *Salmonella* isolates from the food and food environment for *inv* genes found the varying distributions of *invB* (92%), *invE* (96%), and *invH* (54%) genes, while *invA*, *invC*, *invF*, and *invG* were present in all (100%) of the isolates [32]. A study involving 13 *Salmonella* serovars for variations in the composition of SPI-1 to SPI-5 by Southern hybridization reported a highly conserved nature of SPIs in all serovars studied except *Salmonella* Typhimurium which showed variations in SPI-1 to SPI-4 and *Salmonella* Dublin, which showed variations in SPI-5 [33]. Such variations are attributed to insertion or deletion events within the SPIs, which might alter the serovar’s virulence characteristics. Another study comparing the blood and stool isolates of *Salmonella* reported the presence of all the tested genes from SPI-1 to SPI-5, while one or more stool isolates lacked some sequences corresponding to SPI-3 (*misL*) and SPI-4 (*orfL*) [18].

In this study, the *avrA* gene was not detected in *S*. Typhimurium isolates 329, 331, 332, and 334 (Figure-2). The *avrA* gene, which encodes an effector protein, is primarily involved in the enteritis pathway [33] and was detected only in four of the test isolates of this study. In a similar observation, the *avrA* gene was found in 30%–80% of the *Salmonella* isolates from food and food animal environments [34–36]. Genetic rearrangements within the pathogenicity islands result in variations within SPIs. For example, the replacement of a 200 base fragment in the *avrA* region of SPI-1 in three serovars Choleraesuis, Ohio, and Ratchaburi is responsible for the loss of the *avrA* gene and its genetic environment in these serovars [33]. Similarly, deletion of *avrA* gene was observed in SPI-1 of *Salmonella* Enteritidis isolated from poultry and salmonellosis outbreaks [31], as well as in serovars Typhi, Paratyphi, and Arizonae [9]. The absence of the *avrA* gene in all systemic variants of *S. enterica* serotype Paratyphi B has been correlated with the ability of *Salmonella* serovars to evade epithelial defenses, causing severe systemic diseases [37]. Some investigators have observed that the loss of *avrA* increases apoptosis in epithelial cells and macrophages. Moreover, the absence of *avrA* leads to the simultaneous loss of intracellular *Salmonella* carriage and an increase in microbial burden in systemic lymphoid tissues, increasing mortality during the late stages of infection [38]. The loss of *avrA* gene, therefore, is not at the fitness cost of *Salmonella*, and the gene appears to be dispensable.

All gene sequences representing SPI-2 to SPI-4 were detected in all serovars tested in this study, suggesting that these pathogenicity islands are uniformly present in environmental isolates similar to the isolates from food-borne infections. According to a study, SPI-II to SPI-V genes were reportedly well conserved among different *Salmonella* serovars with no significant difference between poultry and outbreak strains [32]. A study comparing environmental isolates of *Salmonella* Newport with clinical *Salmonella* Typhi from a region where salmonellosis is endemic also reported the occurrence of SPI-1 to SPI-6 in 95%–100% of the isolates [39]. These SPIs contribute to the bacterium’s ability to survive the host immune system and spread to new niches within the host. The *stn* gene was also detected in all the isolates tested. Although the role of this enterotoxin in *Salmonella* virulence is unclear, it is speculated to contribute to its enteropathogenicity. Its presence in all serovars of *Salmonella* makes it an ideal target for PCR detection of *Salmonella*. A homologous sequence has been detected in subsp. *bongori* and based on the conserved sequences; a PCR assay to detect both *S*. *enterica* and *Salmonella bongori* has been developed [40].

The prophage-encoded genes *gogB* and *sod*C were absent in non-Typhimurium serovars of *Salmonella* used in the study. The *gogB* gene present in the lysogenic phage Gifsy-1 aids in the colonization of the small intestine by *S*. Typhimurium and its protein product is translocated into host cells through the SPI-2-mediated system [41]. The *sodC* gene located on the prophage encodes a protein that helps in the survival of *Salmonella* inside the macrophages. Although *gogB* and *sodC* were detected in both invasive and enteritis isolates of NTS, they were not uniformly present in all the isolates [42]. While *gogB* gene is always associated with *S*. Typhimurium, as observed in this study, studies have shown considerable variations in the presence of *gogB* and *sodC* genes among other *Salmonella* serovars [43].

## Conclusion

The seafood-borne *Salmonella* do not vary significantly from outbreak strains with respect to the composition of their SPIs. In particular, the SPI-1, the role of which is well established in the pathogenicity of *Salmonella*, is highly conserved in environmental isolates, similar to the observation made by several investigators. The significance of the loss of some SPI and phage-encoded genes on the virulence and environmental persistence of *Salmonella* needs further investigation.

## Authors’ Contributions

ML and SHK: Contributed to the conception and design of the study. PP: Sample preparation. FS: Conducted the experiments, analyzed the data, and drafted the manuscript. ML, SHK, and BBN: Contributed to the interpretation of the data and critical revision of the manuscript. All authors have read, reviewed, and approved the final manuscript.
